# Fabrication of Alkoxyamine-Functionalized Magnetic Core-Shell Microspheres via Reflux Precipitation Polymerization for Glycopeptide Enrichment

**DOI:** 10.3390/polym8030074

**Published:** 2016-03-04

**Authors:** Meng Yu, Yi Di, Ying Zhang, Yuting Zhang, Jia Guo, Haojie Lu, Changchun Wang

**Affiliations:** 1 Department of Macromolecular Science, State Key Laboratory of Molecular Engineering of Polymers, Laboratory of Advanced Materials, Fudan University, Shanghai 200433, China; yumeng@fudan.edu.cn (M.Y.); 11110440007@fudan.edu.cn (Yu.Z.); guojia@fudan.edu.cn (J.G.); 2 Institutes of Biomedical Sciences and Department of Chemistry, Fudan University, Shanghai 200032, China; 14111510007@fudan.edu.cn (Y.D.); ying@fudan.edu.cn (Yi.Z.)

**Keywords:** alkoxyamine-functionalized microspheres, reflux precipitation polymerization, magnetic composite microspheres, oxime click, glycoproteins/glycopeptides enrichment

## Abstract

As a facile method to prepare hydrophilic polymeric microspheres, reflux precipitation polymerization has been widely used for preparation of polymer nanogels. In this article, we synthesized a phthalamide-protected *N*-aminooxy methyl acrylamide (NAMAm-*p*) for preparation of alkoxyamine-functionalized polymer composite microspheres via reflux precipitation polymerization. The particle size and functional group density of the composite microspheres could be adjusted by copolymerization with the second monomers, *N*-isopropyl acrylamide, acrylic acid or 2-hydroxyethyl methacrylate. The resultant microspheres have been characterized by TEM, FT-IR, TGA and DLS. The experimental results showed that the alkoxyamine group density of the microspheres could reach as high as 1.49 mmol/g, and these groups showed a great reactivity with ketone/aldehyde compounds. With the aid of magnetic core, the hybrid microspheres could capture and magnetically isolate glycopeptides from the digested mixture of glycopeptides and non-glycopeptides at a 1:100 molar ratio. After that, we applied the composite microspheres to profile the glycol-proteome of a normal human serum sample, 95 unique glycopeptides and 64 glycoproteins were identified with these enrichment substrates in a 5 μL of serum sample.

## 1. Introduction

In the past decades, multi-functional polymeric microspheres have attracted great attention because of their broad applications in modern science and technology [1]. The particle size and functional groups of polymeric microspheres both play an important role in practical applications. For example, micron-size microspheres are usually applied as chromatographic separation media in size-exclusion chromatography (SEC) [2,3], and nanoscale microspheres are widely used in biomedical fields [4,5]. In order to fulfil different requirements, more and more hybrid microspheres with inorganic cores and polymeric shells have been prepared with different methods, including emulsion polymerization [6,7], surface initiated living polymerization (e.g., SI-ATRP [8,9,10,11], SI-RAFT [12,13]), and so on. The functional microspheres show great potentials in protein enrichment [14,15,16], drug delivery [17,18,19], imaging [20,21], and diagnosis [22,23].

Reflux precipitation polymerization (RPP), which is a heterogeneous polymerization system without addition of any surfactants, has been adopted to prepare a variety of microspheres with hydrophilic shell [24,25]. However, depending on the RPP requirement, the available functional monomers are synthetically challenging. Although the post-modification strategy have found success in this regard [26,27], the procedure is tedious and often low yielding. Therefore, exploration of new functional monomers for RPP is highly required.

Imine is an important chemistry structure, which has been studied for many years [28]. The substituent groups on carbon atom affect the stability of the imine structure, and the electrophilic structures can stabilize imine in aqueous solution. Compared with aliphatic Schiff base, the aromatic Schiff base is more stable [29]. Of imine derivatives, hydrazones are very useful in controllable drug release because of their slow hydrolysis property in weak acidic aqueous solution [30]. Oximes are the most stable imine structure, which have been widely used in bioconjugation [31,32,33]. Recently, it is found that the nucleophilic catalyst could accelerate the reaction between alkoxyamine and ketone/aldehyde groups [34,35,36,37]. This finding implies that alkoxyamine-functionalized nanomaterials have great potentials for use in identification of aldehyde-containing biomolecules, for example, glycopeptides and glycoproteins.

Up to date, some papers report the post-modification preparation of alkoxyamine-functionalized polymers [32,35,38], but they do not concern with the problem of functional group density. In order to directly prepare the alkoxyamine polymers or polymer microspheres, *N*-boc-protected alkoxyamine monomers have been synthesized [33,39], but the deprotection condition is not fit to prepare hybrid materials. Sumerlin and co-workers prepared a phthalamide-protected alkoxyamine monomer, and the well-defined polymer was synthesized via RAFT polymerization [40]. However, it is evident that these monomers are not suitable to polymerize in RPP. 

Within the context, we designed a new alkoxyamine-based monomer to directly prepare alkoxyamine-functionalized polymer microspheres with magnetite nanoclusters in core by the RPP route. Phthalamide-protected *N*-aminooxy methyl acrylamide (NAMAm-*p*) was synthesized as monomer [41], and it was subjected to the RPP for well controlling the particle size and functional group density. Finally, the tailor-made microspheres were applied in enrichment of glycoproteins and glycopeptides.

## 2. Materials and Methods

### 2.1. Materials

Iron(III) chloride hexahydrate (FeCl_3_·6H_2_O), ammonium acetate (NH_4_Ac), sodium acetate anhydrous (NaAc), ethylene glycol, anhydrous ethanol, trisodium citrate dihydrate, aqueous ammonia solution (NH_3_·H_2_O, 25%), *N,N’*-dimethylformamide (DMF), aniline, 2,2-azobisisobutyronitrile (AIBN), *N*-isopropyl acrylamide (NIPAm), acrylic acid (AA), 2-hydroxyethyl methacrylate (HEMA) were purchased from Sinopharm Chemical Reagent Co., Ltd. (Shanghai, China). *N,N′*-Methylene-bis-acrylamide (MBA), *N*-methylolacrylamide, diisopropyl azodicarboxylate (DIPA), triphenylphosphine, *N*-hydroxyphthalimide (NOP), hydrazine monohydrate were purchased from Aladdin (Shanghai, China). Methacryloxypropyltriethoxysilane (MPS), bovine serum albumin (BSA), asialofetuin from fetal calf serum (ASF), myoglobin from horse heart (MYO), lysozyme (LYS), sodium periodate (NaIO_4_), ammonium bicarbonate (NH_4_HCO_3_), urea, MALDI matrix (α-cyano-4-hydroxycinnamic acid, CHCA) were all obtained from Sigma (St. Louis, MO, USA). Acetonitrile (ACN, 99.9%, chromatographic grade) and trifluoroacetic acid (TFA) were purchased from Merck (Darmstadt, Germany). The glycerol free peptide-*N*-glycosidase (PNGase F, 500 units/μL) and SDS-PAGE molecular weight standards (6.5–175 kDa) were from New England Biolabs (Ipswich, MA, USA). Sep-Pak C18 columns were from Waters (Shanghai, China). Human serum was provided by Fudan University Shanghai cancer center and stored at −80 °C before analysis. Water used in experiments was ultrapure water prepared using a Milli-Q50SP Reagent Water System (Millipore, Bedford, MA, USA).

### 2.2. Instrument and Analysis

The polydispersity index (*M*_w_/*M*_n_) of the polymers was measured by gel permeation chromatography (GPC). The GPC anylysis was performed on an Agilent 1100 equipped with a G1310A pump, a G1362A refractive index detector, and a G1315A diode-array detector, poly (methyl methacrylate) (PMMA) standard samples were used as calibration, DMF was used as mobile phase, the measurement condition is at 40 °C with an elution rate of 0.5 mL/min. Transmission electron microscopy (TEM) images were taken on a JEM-2100F transmission electron microscope (JEOL, Tokyo, Japan) at an accelerating voltage of 200 kV. Samples dispersed at an appropriate concentration were cast onto a carbon coated copper grid. Magnetic characterization was carried out on a VSM on a Model 6000 physical property measurement system (Quantum, Blaien, WA, USA) at 300 K. Hydrodynamic diameter (Dh) measurements were conducted by dynamic light scattering (DLS) with a ZEN3600 (Malvern, Malvern, UK) Nano ZS instrument using He–Ne laser at a wavelength of 632.8 nm. Fourier transform infrared spectra (FT-IR) were recorded on a Magna-550 (Nicolet, Waltham, MA, USA) spectrometer. Spectra were scanned over the range of 400–4000 cm^−1^. All of the dried samples were mixed with KBr and then compressed to form pellets. Thermogravimetric analysis (TGA) measurements were performed on a Pyris 1 TGA instrument (PerkinElmer, Waltham, MA, USA). All measurements were taken under a constant flow of atmosphere of 40 mL/min. The temperature was first increased from room temperature to 100 °C and held until constant weight, and then increased from 100 to 600 °C at a rate of 20 °C/min. NMR data were measured by Varian Mercury AS400 NMR System (Varian, Palo Alto, CA, USA). The sodium dodecyl sulfate–polyacrylamide gel electrophoresis (SDS–PAGE) was performed using 4%–15% precast polyacrylamide gels and Mini-Protean Tetra cell (Tanon, Shanghai, China). Protein concentration was obtained by measuring absorbance at 595 nm using BioTek Power Wave XS2 microplate reader (BioTek, Winooski, VT, USA).

### 2.3. Synthesis of N-Aminooxy Methylacylimde-p (NAMAm-p)

In a typical procedure, *N*-methylol acrylimide (10.1 g), *N*-hydroxyphthalimide (16.3 g), triphenylphosphine (26.2 g) were dissolved by 150 mL ACN in a 250 mL three-necked round-bottomed flask. After purging nitrogen for 0.5 h, diisoproply-azodicarboxylate (20.2 g) was dropped slowly into the solution within 1 h and the solution was stirred overnight at room temperature. At last, 5 mL ethanol was added into the solution and the mixture was concentrated under reduced pressure. The product was washed with 50 mL chloroform for 3 times and dried in the vacuum to obtain the pure NAMAm-*p* white powder (16.5 g, 65%). ^1^H NMR (400 Hz, d_6_-DMSO): δ 9.30 (t, H), δ 7.84 (s, 4H), δ 6.10 (qd, 2H), δ 5.67 (dd, H), δ 5.17 (d, 2H). ^13^C NMR: δ 165.20 (CONH), δ 164.78 (CONO), δ 135.20 (COCCH), δ132.56 (CHCH_2_), δ 129.41 (CCHCH), δ 126.51 (CH_2_CH), δ 123.64 (CHCHCH), δ 63.04 (NHCH_2_).

### 2.4. Preparation of PNAMAm-p Polymer Chain and Deprotection for PNAMAm

The PNAMAm polymer was prepared by traditional radical polymerization. Typically, NAMAm-*p* (500 mg) and AIBN (20 mg, 0.122 mmol) were dissolved by 50 mL ACN in a dried 100 mL two-necked flask, followed by 5 min ultra-sonication to ensure the formation of homogeneous solution. After purging nitrogen for 0.5 h, the solution was stirred with magnetic stirring bar and heated to 90 °C, keeping reflux for 4 h. The product was concentrated under reduced pressure, and the residual powder were dissolved in 20 mL DMF and precipitated with methanol twice. At last, the pure PNAMAm-*p* white powder (362 mg, 70%) was obtained. 

For the deprotection, PNAMAm-*p* (300 mg) was dissolved in the solution of 20 mL DMF with 2 mL hydrazine hydrat, then the solution was stirred at room temperature for 2 h. The final product of PNAMAm as white powder were collected by precipitation with methanol and dried at 40 °C in vacuum oven (97 mg, 69%).

### 2.5. Preparation of Monodisperse Crosslinked PNAMAm-p Microspheres and Deprotection Study 

The microspheres preparation was carried out via reflux-precipitation polymerization by varying the crosslinker and monomer concentrations and reaction times. A typical polymerization procedure was as follows: NAMAm-*p* (100 mg), MBA (25 mg) and AIBN (5 mg) were dissolved by 20 mL ACN in a dried 50 mL single-necked flask, followed by 5 min ultra-sonication. The flask was then equipped with an allihn condenser and immersed in oil bath, and the reaction temperature was slowly increased from ambient temperature to 90 °C, the reaction solution kept refluxing for one hour without stirring at this temperture. The final products were collected by centrifugation following by repeated washing with ACN and DMF. The crosslinked PNAMAm-*p* microspheres were deprotected with 10% hydrazine hydrate in DMF solution for 2 h at room temperature. The final microspheres were collected by centrifugation and dried at 40 °C in vacuum oven.

### 2.6. Preparation of MPS Modified MSPs (MSP-m)

The MSPs (magnetic supraparticles) were prepared by a modified solvothermal route [14]. Typically, FeCl_3_·6H_2_O (4.3 g), NaAc (4.8 g), sodium citrate (1.0 g) were dissolved in 70 mL of ethylene glycol. The mixture was stirred vigorously for 1 h at 160 °C to form a homogeneous black solution and then transferred into a Teflon-lined stainless-steel autoclave (100 mL capacity). The autoclave was heated at 200 °C and maintained for 20 h, then it was cooled to room temperature. The black precipitate MSPs were washed twice by ethanol. Then, the MSPs were modified with MPS through a sol–gel method. Typically, all of the products were dispersed in 160 mL ethanol and 40 mL DI water, then 3 mL ammonium hydroxide and 1.2 mL MPS were added into the mixture, and the mixture was stirred overnight at room temperature. The final products were washed twice with ethanol by a magnet, and dried in the vacuum at 40 °C.

### 2.7. Preparation of MSP@PNAMAm-p Magnetic Hybrid Microspheres and Deprotection for MSP@PNAMAm

The MSP@PNAMAm-*p* magnetic composite microspheres were prepared by a modified RPP route under different conditions by varying the monomer and crosslinker concentrations, in all the formulation, the MSP-m concentration was fixed at 1.25 mg/mL. Typically, MSP-m (25 mg), NAMAm-*p* (100 mg), MBA (25 mg), AIBN (5 mg) were dispersed in 20 mL ACN in a dried single-necked flask by 5 min ultra-sonication to ensure the formation of a stable dispersion. The mixture was heated to 90 °C and kept the reaction for 2 h, the final product was collected and washed with DMF twice. The deprotection procedure was the same as above and the final products were dried at 40 °C in the vacuum for further use.

### 2.8. Enrichment of Model N-glycoprotein and N-glycopeptides with MSP@PNAMAm Microspheres

In a typical procedure, 5 μg BSA, 5 μg RNase B and 5 μg LYS were dissolved in 40 μL oxidation acetate buffer (pH = 5.6, 100 mM), and then 10 μL 50 mM NaIO_4_ solution was added. After 1h shaking in the darkness at room temperature, the oxidation process was quenched by 10 μL 50 mM sodium sulfite solution Then, the oxidized products were lyophilized and the coupling buffer (pH = 4.6, containing 10 mM ammonium acetate) were introduced. After the addition of MSP@PNAMAm and 100 mM aniline, the mixture above were kept at 45 °C for 4 h under constant shaking in the dark. Then, the nonspecifically captured peptides were removed by washing twice with 50% DMF and 50 mM NH_4_HCO_3_ sequentially with the aid of magnet. Next, the microspheres were incubated in the mixture containing 1 μL of PNGase F (500 units per μL) and 50 mM NH_4_HCO_3_ at 37 °C overnight, the supernatant and eluate were analyzed by SDS–PAGE individually.

For the enrichment of *N*-glycopeptides, the standard protein (ASF and MYO) were dissolved in 50 mM NH_4_HCO_3_ (pH = 8.0) and denatured by incubating at 100 °C for 10 min. After cooling down to room temperature, trypsin was added to the solution at an enzyme-to-substrate ratio of 1:50 (*w*/*w*) and the proteolysis was proceeded overnight at 37 °C, followed by lyophilization of the digested sample. The lyophilized glycopeptides and non-glycopeptides at different ratios (1:10, 1:50, 1:100) were used as the enrichment sample, and the procedure of the enrichment was similar to that of model proteins. After digestion of the PNGase F, the supernatant and eluate were analyzed by MALDI-TOF. All experiments of standard glycopeptides were performed in reflector positive mode on AB Sciex 5800 MALDI-TOF/TOF mass spectrometer (AB Sciex, Framingham, MA, USA) with a pulsed Nd/YAG laser at 355 nm. 0.5 μL aliquot of the eluate and 0.5 μL of CHCA (10 mg/mL CHCA in 50% ACN containing 0.1% TFA) matrix were spotted onto a MALDI target plate.

### 2.9. Enrichment of N-glycopeptides from Human Serum with MSP@PNAMAm

In order to evaluate the enrichment capability of the MSP@PNAMAm magnetic hybrid microspheres, we further used MSP@PNAMAm to enrich glycopeptides in human serum from normal volunteers. The tryptic digest of 5 μL human serum was treated according to the above procedure after reduction and alkylation. Then the eluate was collected and sent for nano-LC-MS/MS analysis. The human serum protein sample was analyzed by a LC-20AD system (Shimadzu, Tokyo, Japan) connected to a LTQ orbitrap mass spectrometer (Thermo Electron, Bremen, Germany) equipped with an online nanoelectrospray ion source (Michrom Bioresources, Auburn, CA, USA). The lyophilized deglycosylated peptides were redissolved in solution containing 5% ACN containing 0.1% FA. Then the sample solution was loaded on a CAPTRAP column (0.5 mm × 2 mm, MICHROM Bioresources, Auburn, CA, USA) in 4 min with a flow rate of 20 μL/min. For a gradient separation, the gradient elution was performed as follows: Acetonitrile from 5% to 45% (95% ACN in 1% FA) over 100 min at a flow rate of 500 nL/min. For each cycle of duty, full mass scan was acquired from 400 to 2000 *m*/*z*. The MS/MS spectra were obtained in data-dependent ddMS2 mode. The 12 most intense ions with charge 2, 3 or 4 were selected for the MS/MS run, and the a dynamic exclusion duration was 90 s. Finally, three parallel enrichment operations were performed as technical repeats.

### 2.10. Data Analysis

For the enrichment of glycopeptides in the human serum, the raw data derived from the ESI MS/MS analysis was searched by MASCOT, against a database (uniprot. Human). The parameters of the search were set as follows: Enzyme of trypsin (partially enzymatic, two missed cleavages were allowed). Fixed modifications of carboxamidomethylation (C, 57.02150), variable modifications of oxidation (M, 15.99492) and deglycosylation (N, 0.98402). 20 ppm error tolerance of precursor mass and 1 Da offragment mass for the Mascot search. Significance threshold was controlled as p value below 5%. Only peptides’ sequence containing N-X-S/T(XX-S were considered as *N*-linked glycolpeptides.

## 3. Results and Discussion

### 3.1. Polymerization of PNAMAm-p and the Deprotection Study

**Scheme 1:**
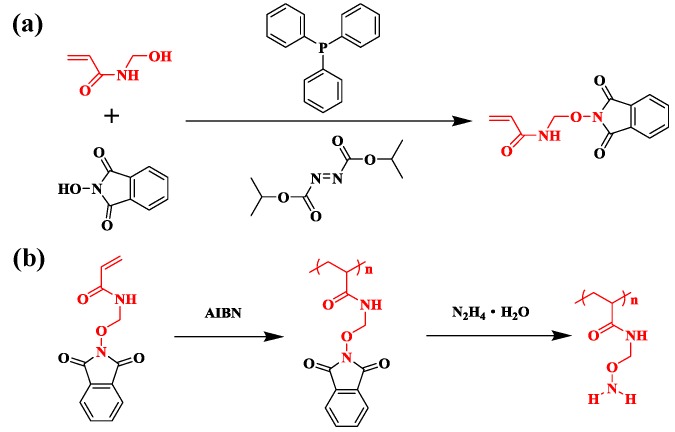
Synthesis of (**a**) monomer NAMAm-*p* and; (**b**) polymer PNAMAm.

**Figure 1:**
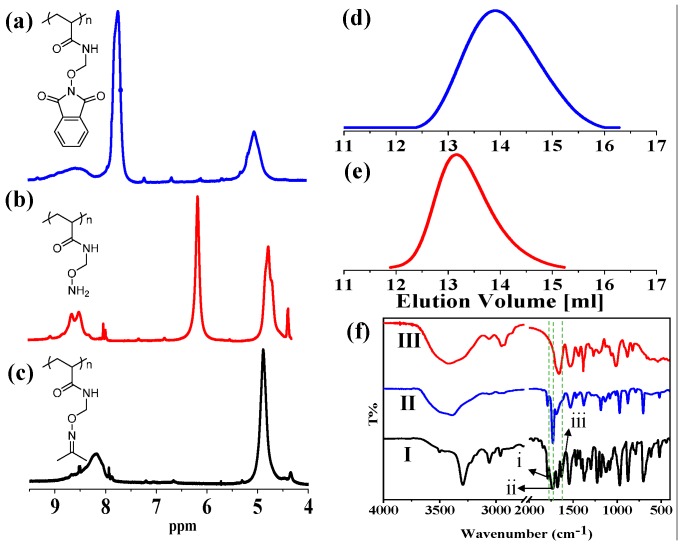
^1^H NMR spectra of (**a**) PNAMAm-*p*; (**b**) PNAMAm, and; (**c**) PNAMAm-m; GPC spectra of; (**d**) PNAMAm-*p* (PDI = 1.36), and; (**e**) PANAMAm (PDI = 1.37); (**f**) FT-IR spectra of (I) NAMAm-*p*; (II) PNAMAm-*p*; and (III) PNAMAm. The labelled peaks are (i) 1790 cm^−1^; (ii) 1735 cm^−1^ and (iii) 1633 cm^−1^.

The monomer NAMAm-*p* was synthesized via Mitsunobu reaction (Scheme 1a) and its molecular structure was confirmed by ^1^H NMR (Figure S1a, Supplementary Material). Then the PNAMAm was prepared by traditional free radical polymerization of NAMAm-*p* and one-step deprotection (Scheme 1b). Compared with the ^1^H NMR spectrum of NAMAm-*p*, the PNAMAm-*p* (Figure 1a) gives the peaks at 9.30, 5.17 and 7.84 ppm, which could be ascribed to secondary amine, methylene and phthalimide, respectively, while the peaks of the –CH=CH_2_ protons (6.10 and 5.67 ppm) was not observed. The result confirmed the obtained PNAMAm-*p* structure, and also, it well agreed with that found in the FT-IR spectra, wherein the stretching peaks of C=C at 1633 cm^−1^ disappeared due to the polymerization of NAMAm-*p* monomer (Figure 1f–I, II).

Deprotection of the PNAMAm-*p* was accomplished by hydrazine hydrate in DMF at room temperature for 2 h. As shown in Figure 1b, the phthalimide peak at 7.84 ppm disappears, the methylene peak shifts from 5.17 to 4.50 ppm, and a new peak is found at 5.90 ppm, which is attributed to the –O–NH_2_ protons. The integral area ratio of peaks at 4.50 and 5.90 ppm is about 1:1, which is well consistent with the theory value. The results reveal that the PNAMAm-*p* is completely deprotected and the alkoxyamine groups are formed. Again, FT-IR spetra (Figure 1f) proved the deprotection due to disappearance of the stretching peaks of C=O at 1790 cm^−1^, C=C at 1735 cm^−1^. The high efficient deprotection ensures the precise control of functional group density on the polymers. According to GPC measurement (Figure 1d), the *M*_n_ of PNAMAm-*p* is 3630 and the *M*_w_ is 3940, the PDI is 1.36. A small difference in PDI (Figure 1d,e) was found, implying the molecular weight distribution of polymer chains did not change much in the deprotection process, and only the side groups were changed. However, the GPC results could not provide more information because the polymer polarity was greatly changed.

We estimiated the reactivity of the side alkoxyamine groups by the model reaction. Acetone was used to react with PNAMAm to form oxime bonds. As shown in Figure 1c, the peak of –O–NH_2_ protons at 5.90 ppm disappears, and the peak of methylene protons shifts from 4.50 to 4.90 ppm. This change is owing to the formation of oxime bonds. The product yield, which could be calculated by comparing the integration of the peak at 4.90 ppm, was about 95%. It means that the acetone is efficiently linked to the PNAMAm under the mild conditions. The results demonstrate that the PNAMAm not only possesses a high reaction activity, but also provides possibility for bioconjugation with aldehyde/ketone-based glycoproteins and glycopeptides in complex physiological environment.

### 3.2. Preparation of PNAMAm Microspheres and MSP@PNAMAm Core-Shell Microspheres

**Scheme 2:**
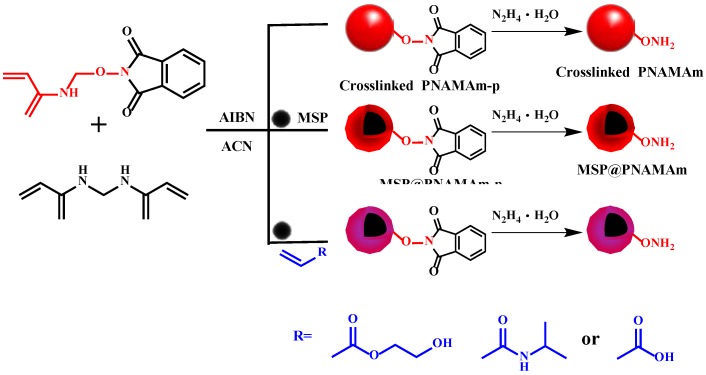
Preparation of the crosslinked PNAMAm micropsheres, MSP@PNAMAm core–shell microspheres, and the core-shell microspheres with varying functional groups (MSP@PNAMAm-R).

To satisfy different potential applications, we studied the copolymerization of the monomer NAMAm-*p* with crosslinker and other monomers to fabricate different kinds of alkoxyamine functional microspheres. The detailed design was shown in Scheme 2. First, we investigated the effect of crosslinker species and relative amounts on the microsphere properties. The three often used crosslinkers, divinylbenzene (DVB), ethylene glycol dimethylacrylate (EGDMA) and *N,N′*-methylene bisacrylamide (MBA), were utilized for the preparation of PNAMAm-*p* microspheres. The results showed that only MBA could afford the corresponding microspheres, the possible reason is related to the higher reactivity of MBA. 

**Figure 2:**
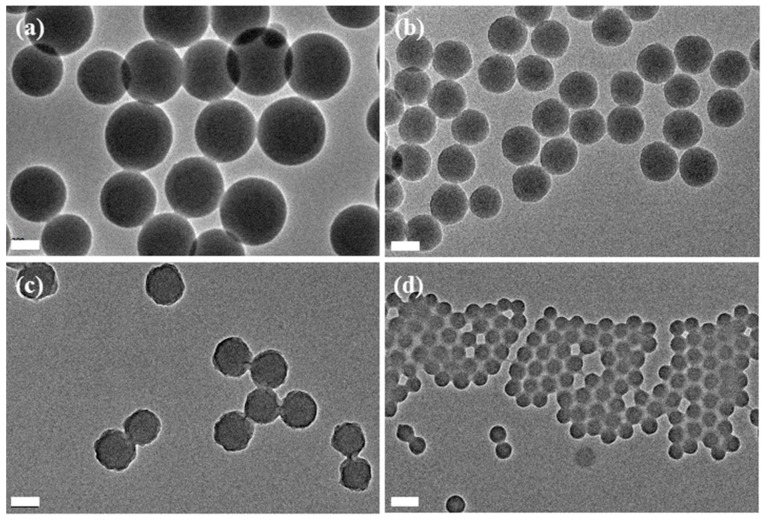
TEM images of PNAMAm with different amount of MBA as crosslinker: (**a**) 20%; (**b**) 30%; (**c**) 40%; (**d**) 50%. The scale bar is 200 nm.

TEM images in Figure 2 display that the particle size is greatly influenced by the feed amount of crosslinker MBA. When 20 wt % MBA was used, the particle size was about 300 nm. With the increase of MBA content to 30 wt % and 50 wt %, the particle sizes accordingly decreased to 200 and 150 nm. Meanwhile, the amount of alkoxyamine groups in the microspheres were reduced (Figure S2, Supplementary Material). When the MBA content was less than 20 wt %, no microspheres were formed.

**Figure 3:**
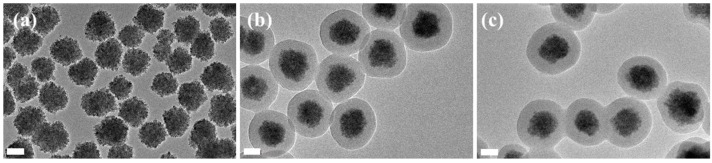
TEM images of (**a**) MSP (magnetic supraparticle); (**b**) MSP@PNAMAm-*p*; (**c**) MSP@PNAMAm. The scale bar is 100 nm.

On the basis of the above results, the core–shell magnetic microsphere consisting of a crosslinking PNAMAm-*p* in shell (20% MBA) and a magnetite supraparticle (MSP) in core was successfully prepared (Figure 3). The characteristic peaks at 1790 and 1735 cm^−1^ were found in the FT-IR spectrum for the MSP@PNAMAm-*p*, proving the formation of PNAMAm-*p* component (Figure S3, Supplementary Material). After the deprotection, the MSP@PNAMAm was obtained, and the core-shell structure was preserved, without any change in morphology (Figure 3c). 

**Figure 4:**
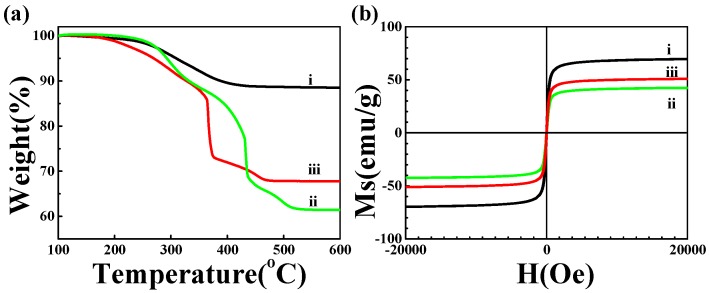
(**a**) TGA and; (**b**) VSM curves of (i) MSP; (ii) MSP@PNAMAm-*p*; (iii) MSP@PNAMAm.

Meanwhile, TGA results were applied to analyze the compositions, and the density of alkoxyamine group (*d*) could be calculated by the formula below:

*d* = (*W*_1_ − *W*_2_)/(*W*_2_ × *M*_pt_) (1)

where, *W*_1_ and *W*_2_ represent the final residual weight percentages of the core-shell composite microspheres after and before the deprotection at 600 °C in air; *M*_pt_ is 132 g/mol, which is the differ molecular weight caused by deprotection. The microspheres remained 65.4% mass and 78.3% mass at 600 °C before and after deprotection, respectively (Figure 4a). According to the formula above, the alkoxyamine group density was calculated to be 1.49 mmol/g (More details about the calculation are provided in Supplementary Material).

The VSM results also could give the mangetic contents of the resultant microspheres, which coincided with that obtained from the TGA results. Besides, the magnetic hysteresis curves proved that the supraparamagnetic characteristic was unchanged during the deprotection process (Figure 4b).

**Figure 5:**
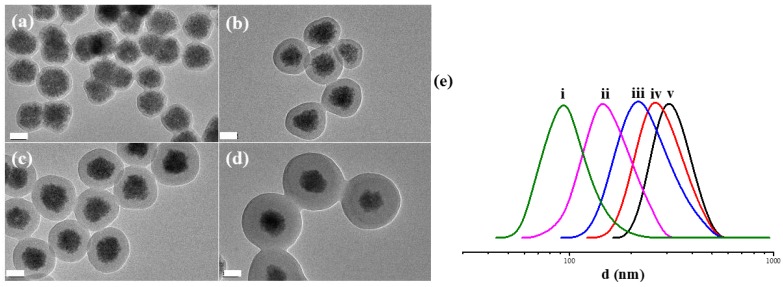
TEM images of MSP@PNAMAm with different thickness of polymeric shell prepared with different solid contents of (**a**) 0.25%; (**b**) 0.375%; (**c**) 0.5%; (**d**) 0.625%, the scale bar is 100 nm; (**e**) DLS results of (i) MSP and MSP@PNAMAm prepared with different solid contents; (ii) 0.25%; (iii) 0.375%; (iv) 0.5%; (v) 0.625%.

In our experiment, we found that the solid content played a key role in adjusting the thickness of the polymer shell (Figure 5). When the solid content was 0.25%, only ~5 nm polymer shell could be obtained. When the solid content was 0.375%, the shell thickness increased to 30 nm. With continuous increase of the solid content to 0.675%, the shell thickness could be changed to almost 100 nm. This increment trend was also confirmed by DLS (Figure 5e). 

In order to quantitatively measure the density of alkoxyamine group in the shell of magnetic composite microspheres, a series of samples with different crosslinking densities were tested by TGA (Table 1). According to the analysis results, the magnetic composite microspheres, which were prepared with different feeding ratios of NAMAm-*p* and MBA and with the same solid content (0.5%), had almost similar shell thickness (Figure S4, Supplementary Material). Through the comparison of the weight changes before and after deprotection, the alkoxyamine group densities were calculated to be 1.49, 1.12, 0.95 and 0.77 mmol/g for the MSP@PNAMAm-1, MSP@PNAMAm-2, MSP@PNAMAm-3, and MSP@PNAMAm-4, respectively.

**Figure 6:**
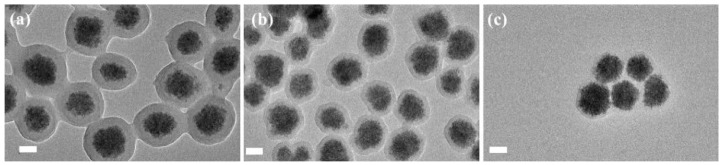
TEM images of (**a**) MSP@PNAMAm-*p*-*co*-PNIPAm; (**b**) MSP@PNAMAm-*p*-*co*-PAA; (**c**) MSP@PNAMAm-*p*-*co*-HEMA. The scale bar is 100 nm.

In addition, the copolymerization properties of NAMAm-*p* were also investigated. NIPAm, AA, and HEMA were used as the second monomers. From the TEM images in Figure 6, the monomer NIPAm has the best performance in copolymerization with NAMAm-*p*, resulting in a uniform and thick polymer shell. The relative ratio of PNAMAm-*p* and PNIPAm could be adjusted by varying the feeding monomer ratios, which was also confirmed by FT-IR (Figure S5, Supplementary Material). Since the monomers of AA and HEMA tend to polymerize on their own, the resultant polymer shells were very thin (Figure 6b,c), but FT-IR spectra (Figures S6 and S7, Supplementary Material) validated that the shells were composed of PNAMAm-*p*-*co*-PAA and PNAMAm-*p*-*co*-HEMA copolymers, respectively.

### 3.3. Selective Enrichment of Glycoproteins and Glycopeptides

**Scheme 3:**
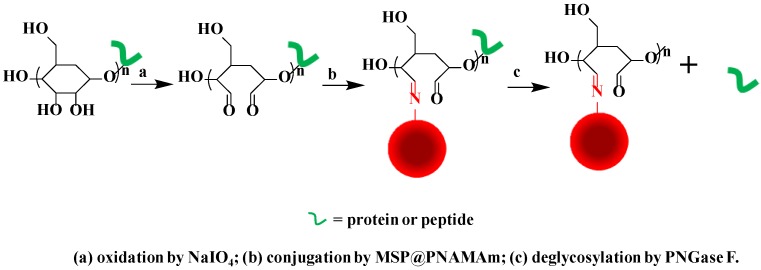
Mechanism of glycoprotein and glycopeptide enrichment with magnetic core–shell microspheres (MSP@PNAMAm-1).

**Figure 7:**
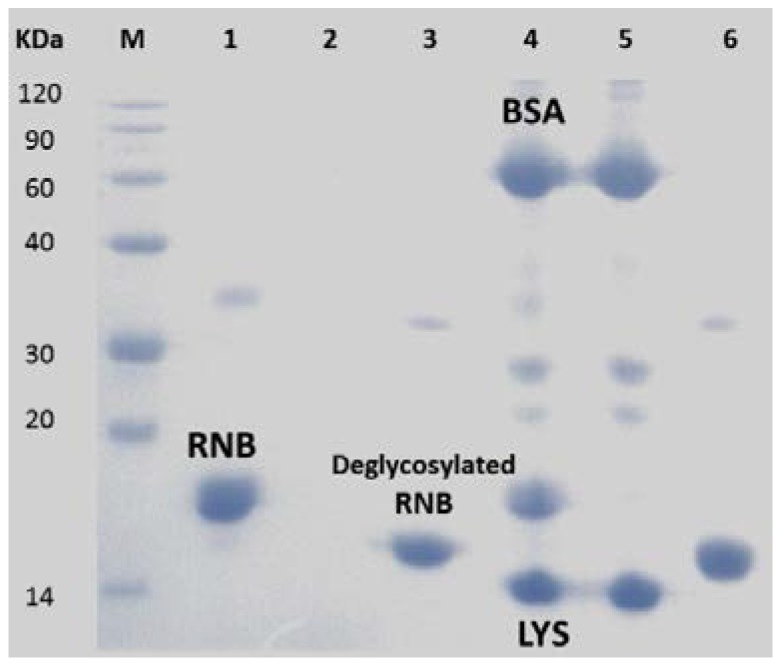
SDS–PAGE analysis of the model glycoprotein proteins before and after treatment with MSP@PNAMAm-1 core-shell microspheres. M stands for protein marker; Lane 1 represents the RNase B; Lane 2 represents the supernatant after enrichment with MSP@PNAMAm-1; Lane 3 represents the released deglycoslated RNB after enrichment; Lane 4 represents the protein mixture of BSA, RNB(RNase B)and LYS(The amount of BSA:RNB:LYS = 1:1:1); Lane 5 represents the supernatant of the protein mixture after enrichment; Lane 6 represents the released deglycosylated RNB after enrichment.

In order to prove the high reactivity between alkoxyamine and aldehyde groups, we evaluated the ability of MSP@PNAMAm to enrich the model glycoproteins. In general, for glycoproteins, the diol structure of saccharide can be oxidized to aldehyde groups, which can bond with the alkoxyamine group. RNB, a typical mono-*N*-glycosylation protein was chosen as the model glycoprotein and the MSP@PNAMAm-1 as the enrichment substrate. The mechanism of enrichment was shown in Scheme 3 and the results were shown in Figure 7. In Lane 1, it was the typical band of protein RNB. After oxidation and conjugation, all of the RNB disappeared in the supernatant (Lane 2). Then with the catalysis of PNGase F, the deglycosylated RNB was eluted in Lane 3. The glycoprotein band with the different molecular weight from Lane 1 represented the lost saccharide structure of RNB by PNGase F. The selectivity of glycoprotein was investigated subsequently by enriching glycoproteins in a mixture with non-glycoproteins (BSA and LYS). The results were shown in Figure 7, Lane 4 to Lane 6. Before enrichment, all the proteins were shown in Lane 4. After oxidation and conjugation, BSA and LYS still remained in the supernatant, but the RNB disappeared in Lane 5. In Lane 6, only deglycosylated RNB was found. There results prove that the MSP@PNAMAm could be applied to enrich glycoproteins selectively, and this property provides a potential ability to enrich glycoproteins and glycopeptides selectively in a complex system. 

**Figure 8:**
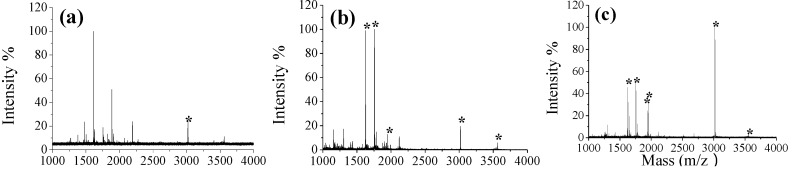
MALDI-TOF mass spectra of the tryptic digest mixture of ASF (asialofetuin) and MYO (myoglobin), the mole ratio of ASF:MYO = 1:10. (**a**) direct analysis; (**b**) analysis after enrichment by MSP@NAMAm-1 and deglycosylation by PNGase F; (**c**) analysis after enrichment by MSP@NAMAm-4 and deglycosylation by PNGase F. The symbols * denote the deglycosylated peptides.

Meanwhile, the selective enrichment of glycopeptides was also investigated from the digests of the mixture containing ASF and MYO (ASF:MYO = 1:10). Considering the effect of functional group density, MSP@PNAMAm-1 and MSP@PNAMAm-4, which had 1.49 and 0.77 mmol/g of alkoxyamine groups, respectively, were applied in glycopeptides enrichment, and the results were shown in Figure 8. Before enrichment, the dominant peaks in the spectrum were due to non-glycopeptides. After enrichment with the two samples, all the dominant peaks (*m*/*z* values of 1627.6, 1755.7, 1950.8 and 3017.4) were attributed to deglycopeptides from the digest of ASF. What is more, it was interesting that the glycopeptide distribution in the mass spectrum were affected by the enrichment materials. For the MSP@PNAMAm-4, the strongest peak was at 3017.4 (*m*/*z*), while the intensity of this peak was weaker than those at 1627.6 (*m*/*z*) and 1755.7 (*m*/*z*) with MSP@PNAMAm-1. In addition, it was observed when non-glycopeptide was mixed with glycopeptides in a high molar ratio (100:1) (Figure S8, Supplementary Material). The early reports found the same phenomenon [29,42]. In consideration of the complex conjugation-elution process, it is difficult to explain this phenomenon now, but it is sure that different surface structures have varying interaction with glycopeptides, and the difference might affect the enrichment results. 

The enrichment ability of MSP@PNAMAm-1 and MSP@PNAMAm-4 were further investigated by profiling the *N*-glycoproteome of a normal human serum sample. We found that the result of MSP@PNAMAm-1 was not good. In contrast, MSP@PNAMAm-4 with the low functional group density showed much better enrichment ability, 95 unique glycopeptides and 64 glycoproteins were identified in a 5 μL serum sample (Table S1, Supplementary Material). It implies that a suitable functional group density is benificial for glycoproteins and glycopeptides enrichment in complex physiological environment. In addition, it proves that the MSP@PNAMAm microspheres exhibit a great potential in real biomedical application.

## 4. Conclusions

In this paper, we have prepared the alkoxyamine-functionalized magnetic core-shell microspheres via reflux precipitation polymerization. Alkoxyamine acrylamide monomer with a protective group phthalamide was synthesized, and underwent the reflux precipitation polymerization for alkoxyamine-functionalized polymer microspheres. By varying a series of reaction conditions including monomer concentration, comonomer species and crosslinker content, the core-shell magnetic composite microspheres were constructed to achieve the tunable shell thickenss, controllable alkoxyamine group density, and versatile copolymer composition. After the deprotection, the MSP@PNAMAm displayed high activity to couple with carbonyl compounds under mild conditions. They could serve as enrichment substrate to efficiently identify glycoprotein/glycopeptides. Moreover, we found success in profiling the glycoproteome in human serum samples with the structure-optimized composite microspheres. 

## Figures and Tables

**Table 1:** Recipe, TGA data and the calculated alkoxyamine group density of the MSP@PNAMAm samples with different feeding amount of MBA.

Sample	*m*_(MSP)_ mg	*m*_(NAMAm-*p*)_ mg	*m*_(MBA)_ mg	*W*_1_	*W*_2_	*D* mmol/g
MSP@PNAMAm-1	25	80	20	78.30%	65.40%	1.49
MSP@PNAMAm-2	25	75	30	72.40%	63.10%	1.12
MSP@PNAMAm-3	25	60	40	76.50%	67.96%	0.95
MSP@PNAMAm-4	25	50	50	73.80%	67.00%	0.77

## Supplementary Materials

Supplementary materials can be found at www.mdpi.com/2073-4360/8/3/74/s1.
