# Supplementary material for: Fabrication of Alkoxyamine-Functionalized Magnetic Core-Shell Microspheres via Reflux Precipitation Polymerization for Glycopeptide Enrichment
Source: Polymers (Basel). 2016 Mar 4;8(3):74. doi: 10.3390/polym8030074 (PMC6432552; doi:10.3390/polym8030074)
Supplement: Supplementary file 1 [file polymers-08-00074-s001.pdf]

Supplementary Materials: Fabrication of Alkoxyamine-Functionalized Magnetic Core–Shell Microspheres via Reflux Precipitation Polymerization for Glycopeptide Enrichment

Meng Yu, Yi Di, Ying Zhang, Yuting Zhang, Jia Guo, Haojie Lu and Changchun Wang

The density of alkoxyamine group can be calculated according to TGA results. For the MSP@PNAMAm and MSP@PNAMAm, the final residual weight percentages of both two samples in 600 °C represented the inorganic magnetic core. For 1 g MSP@PNAMAm, we can use W_1/W_2 to represent the corresponding MSP@PNAMAm mass, and the differ $(W_1 - W_2)/W_2$ was caused by the deprotection, the loss of phthalimide group. So the mole of phthalimide in 1 g MSP@PNAMAm can be calculated by the formula below:

$$d = (W_1 - W_2)/(W_2 \times M_{pt}) \quad (1)$$

Where M_{pt} is the differ molecular weight caused by deprotection, the value is 132 g/mol. The d value could also represent the density of alkoxyamine group.

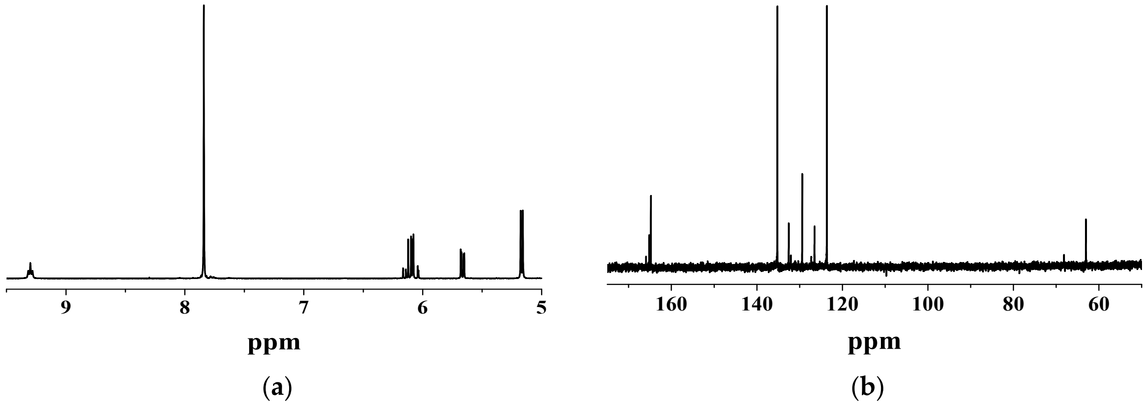

Figure S1. (**a**) ^{1}H NMR and (**b**) ^{13}C NMR spectra of monomer NAMAm-*p*.

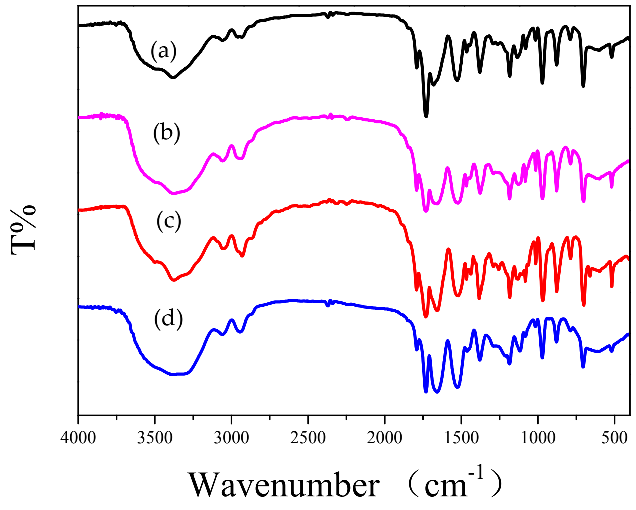

Figure S2.FT-IR spectra of PNAMAm-*p* microspheres prepared with different ratios of MBA; (**a**) 20%; (**b**) 30%; (**c**) 40%; (**d**) 50%.

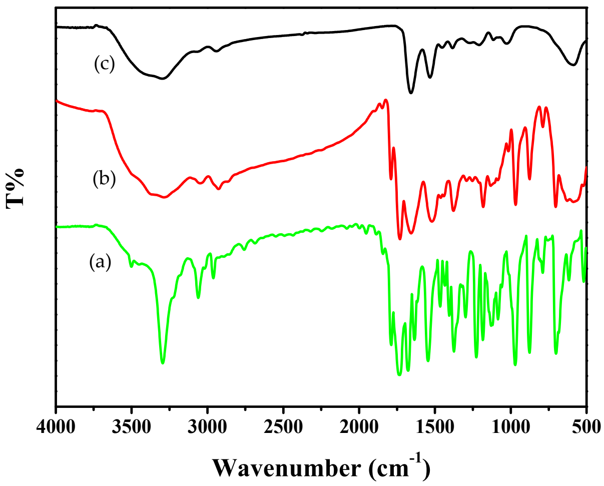

Figure S3. FT-IR spectra of (**a**) NAMAm-*p*, (**b**) MSP@PNAMAm-*p* and (**c**) MSP@PNAMAm.

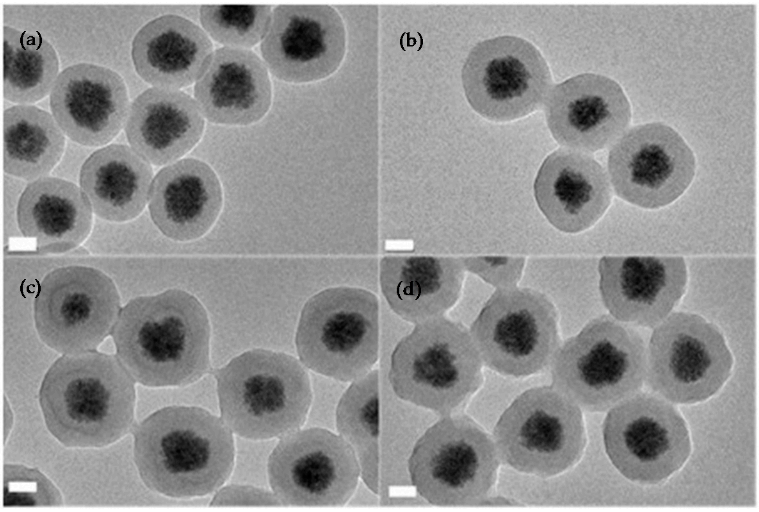

Figure S4.TEM images of MSP@PNAMAm-*p* with different ratios of NAMAm and MBA: (**a**) 4:1; (**b**) 7:3; (**c**) 3:2, (**d**) 1:1. The scale bar is 100 nm.

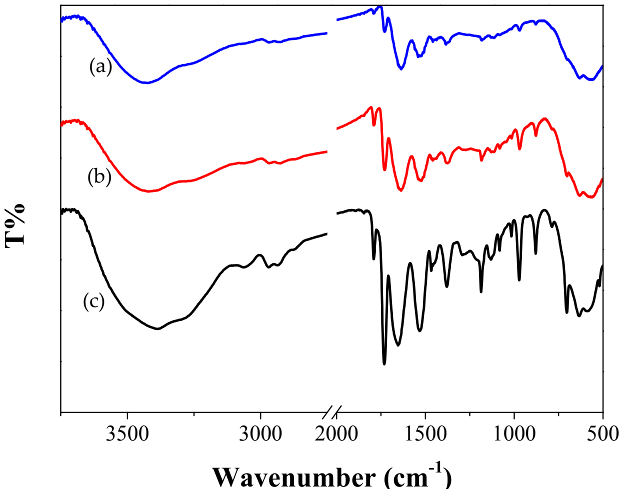

Figure S5. FT-IR Spectra of MSP@PNAMAm-*p*-*co*-PNIPAm with different ratios of NAMAm-*p* and NIPAm: (**a**) NAMAm-*p*:NIPAm = 1:3; (**b**) NAMAm-*p*:NIPAm = 1:1; (**c**) NAMAm-*p*:NIPAm = 3:1

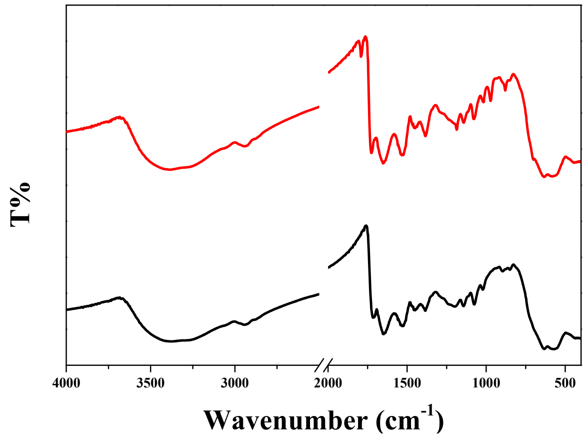

Figure S6. FT-IR Spectra of (**a**) MSP@PNAMAm-*p*-*co*-PAA and (**b**) MSP@PNAMAm-*co*-PAA.

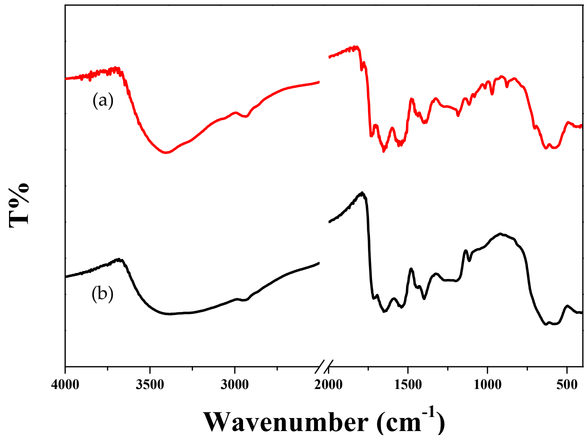

Figure S7. FT-IR Spectra of (**a**) MSP@PNAMAm-*p*-*co*-PHEMA and (**b**) MSP@PNAMAm-*co*-PHEMA.

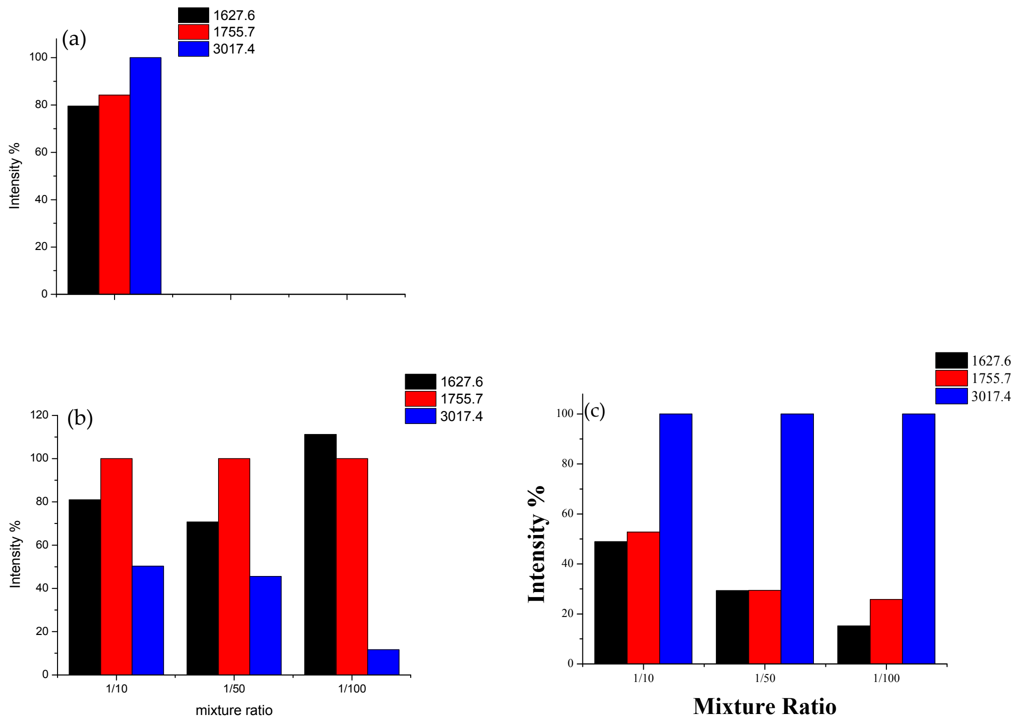

Figure S8. Relative intensity of different signal peaks. (**a**) tryptic digest of ASF following deglycosylation by PNGase F; (**b**) enriched by MSP@PNAMAm-1 in different mixture; (**c**) enriched by MSP@PNAMAm-4 in different mixture.

Table S1. List of identified glycoproteins from 5μL human serum after solid phase extraction with MSP@PNAMAm after three parallel runs, N# denotes the *N*-linked glycosylation site.

NO.	Protein	Description	Peptide
1.	Q9UQM7|KCC2A_HUMAN	Calcium/calmodulin-dependent protein	N#FSGGKSGGNK
2.	Q9ULV1|FZD4_HUMAN	Frizzled-4	ISMCQNLGYN#VTK
3.	Q9P2D1|CHD7_HUMAN	Chromodomain-helicase-DNA-binding protein	EN#ATNGVQQLSK<br>EESMEIHATGKHSESNAELGQLYWPN#TSTLTTR
4.	Q9P225|DYH2_HUMAN	Dynein	QNN#VSVDSLSWEFIVSTVDDSNLVYPPK
5.	Q9NZR2|LRP1B_HUMAN	lipoprotein	SAEQSCN#SSFFMCKNGR
6.	Q9NUQ2|PLCE_HUMAN	acyltransferase	LYCVYQSMVLFFFEN#YTGVQILLYGDLPKNKENIIYLANHQSTVDWIVADILAIR
7.	Q96RI8|TAAR6_HUMAN	amine-associated receptor	MSSN#SSLLVAVQLCYANVN#GSCVKIPFSPGSR
8.	Q96PY0|K1908_HUMAN	Uncharacterized protein	GCGDSGSSGMAQRAQAGSN#QSRGK
9.	Q96MT8|CEP63_HUMAN	Centrosomal protein	AN#DTICANELEIER
10.	Q92752|TENR_HUMAN	Tenascin-R	GTN#ESDSATTQFTTEIDAPK
11.	Q8WXI2|CNKR2_HUMAN	Connector enhancer	TLSHKLN#ASAK
12.	Q8WVX9|FACR1_HUMAN	Fatty acyl-CoA	ASNNALADLVPVDVVVN#MSLAAAWYSGVNRPR
13.	Q8NHS9|SPT22_HUMAN	Spermatogenesis-associated protein	DGNKN#TSLK<br>IPEPPN#LSRNK<br>DGNKN#TSLKTWNK
14.	Q8NF91|SYNE1_HUMAN	Nesprin-1	VSQLSSQYLALSN#LTK
15.	Q8NE71|ABCF1_HUMAN	ATP-binding cassette	AANAAENDFSVSQAEMSSRQAMLEN#ASDIK
16.	Q8N9W8|FA71D_HUMAN	Protein FAM71D	KN#TSKTTMR<br>FLPLQFVTLSVHDAEN#MSLK
17.	Q8N3K9|CMYA5_HUMAN	Cardiomyopathy-associated protein	SNYAQFISN#TSASNADKMVSNKEMPK<br>IEAFVSEIESFFNTIEEN#CSKNEKR
18.	Q8JTG9|L_ABLVH	Large structural protein	LLN#YTLGNR<br>VGGLAAQAMISLWLHGEHSESN#RSRK

Table S1. *Cont.*

NO.	Protein	Description	Peptide
19.	Q8IVL0|NAV3_HUMAN	Neuron navigator	SSPVTVN#QTDK<br>EPTKIGSGRSSPVTVN#QTDK
20.	Q86U42|PABP2_HUMAN	Polyadenylate-binding protein	QMN#MSPPPGNAGPVIMSIEEK
21.	Q6TFL3|CC171_HUMAN	Coiled-coil domain-containing protein	MNLN#TSSNTGDTQR
22.	Q6SW37|EP84_HCMVM	Early phosphoprotein	N#NTRGGGGGGGGGRNSR
23	Q6P1J6|PLB1_HUMAN	Phospholipase B1	VLVNLVDFLN#PTIMR
24	Q2KJY2|KI26B_HUMAN	Kinesin-like protein	EDN#GSEGQLTNREGPELPASKMQR<br>CSSGHGSDN#SSVLSGELPPAMGKTALFYHSGGSSGYESVMRDSEATGSASSAQDSTSEN#SSSVGGR
25.	Q16827|PTPRO_HUMAN	Receptor-type tyrosine-protein	QHRTAPYPPQN#ISVR
26.	Q16821|PPR3A_HUMAN	Protein phosphatase 1	EVLDDNANPAHGN#GTVQIPCPSSDQLMAGNLNK
27.	Q14558|KPRA_HUMAN	Phosphoribosyl pyrophosphate synthase-associated protein	N#ATVHPGLELPLMMAK
28	Q14527|HLTF_HUMAN	Helicase-like transcription	AGGVGLN#LSAASR<br>EYNVNDDSMKLGGN#NTSEKADGLSK<br>QICCHTYLLTNAVSSNGPSGN#DTPEELRKK
29	Q13901|C1D_HUMAN	Nuclear nucleic acid-binding protein	N#ASKVANKGK
30	Q13023|AKAP6_HUMAN	A-kinase anchor protein	N#GSDSLQRSTSLESWLTSYK<br>TFTGMQNAKQLSLLSHSSSIESLSPGGDLFGLGIFKN#GSDSLQR
31.	Q12913|PTPRJ_HUMAN	Receptor-type tyrosine-protein	IHVAGETDSSNLN#VSEPR<br>TNAIQVFDVTAVN#ISATSLTLIWKVSDN#ESSSNYTYK
32.	Q12879|NMDE1_HUMAN	Glutamate receptor	INN#STNEGMNVK<br>FVKIN#NSTNEGMNVK<br>KKSPDFN#LTGSQSNMLK
33.	Q06187|BTK_HUMAN	Tyrosine-protein	N#GSLKPGSSHRK<br>EGSMSEDEFIEEAKVMMN#LSHEK

Table S1. *Cont.*

NO.	Protein	Description	Peptide
34.	P56524丨HDAC4_HUMAN	Histone deacetylase 4	ILIVDWDVHHGN#GTQQAFYSDPSVLYMSLHR
35.	P49792丨RBP2_HUMAN	E3 SUMO-protein	N#GSLRNADSEIKHSTPSPTR<br>N#VSGISFTENMGSSQQKNSGFRR<br>EMLNSVMQELEDYSEGGPLYKN#GSLRNADSEIK
36.	P32004丨L1CAM_HUMAN	Neural cell adhesion	HQMAVKTN#GTGR<br>YFCLAANDQNN#VTIMANLKVK
37.	P20936丨RASA1_HUMAN	Ras GTPase-activating protein	EPVPMQDQEQVLN#DTVDGKEIYNTIRR
38	P20592丨MX2_HUMAN	Interferon-induced GTP-binding protein	SVMNVVRN#LTYPLK
39	P20273丨CD22_HUMAN	B-cell receptor	RTQSQQGLQEN#SSGQSFFVRNK
40.	P19827丨ITIH1_HUMAN	Inter-α-trypsin	DKICDLLVANNHFAHFFAPQN#LTNMNK
41.	P09871丨C1S_HUMAN	Complement C1s	VKNYVDWIMKTMQEN#STPR
42.	P05877丨ENV_HV1MN	Envelope glycoprotein	N#TTNTNNSTANN#NSNSEGTIK<br>N#TTNTN#NSTANN#NSNSEGTIKGGEMK
43	P05155丨IC1_HUMAN	C1 inhibitor	VGQLQLSHN#LSLVILVPQNLK
44	P04196丨HRG_HUMAN	Histidine-rich glycoprotein	VEN#TTVYYLVLDVQESDCSVLSR
45.	P04114丨APOB_HUMAN	Apolipoprotein	SYN#ETKIKFDK<br>LNGESNLRFN#SSYLQGTNQITGR
46	P02790丨HEMO_HUMAN	Hemopexin	SWPAVGN#CSSALR
47	P02787丨TRFE_HUMAN	Serotransferrin	QQQHLFGSNVTDCSGNFCLFR<br>ILRQQQHLFGSNVTDCSGNFCLFR
48	P02749丨APOH_HUMAN	β-2-glycoprotein	VYKPSAGN#NSLYR<br>MEILDNN#WTALLELWDERHR<br>ELAVN#KTQLENIQKTGQEMIEGGHYASDN#VTTR
49	P01876丨IGHA1_HUMAN	Ig α-1	LAGKPTHVN#VSVVMAEVDGTCY<br>LSLHRPALEDLLLGSEAN#LTCTLTGLR

Table S1. *Cont.*

NO.	Protein	Description	Peptide
50	P01871|C1S_HUMAN	Ig mu	YKN#NSDISSTR
51	P01857|IGHG1_HUMAN	Ig γ	EEQYN#STYR<br>EEQYN#STYRVVSVLTVLHQDWLNGK
52	P01024|CO3_HUMAN	Complement C3	TVLTPATNHMGN#VTFTIPANR
53	P01023|A2MG_HUMAN	α-2-macroglobulin	VSNQTLSLFFTVLQDVPVR
54	P01011|AACT_HUMAN	α-1-antichymotrypsin	TLNQSSDELQLSMGNAMFVKEQLSLLDR
55	P01009|A1AT_HUMAN	α-1-antitrypsin	YLGNATAIFFLPDEGK
56	P00738|HPT_HUMAN	Haptoglobin	NLFLN#HSEN#ATAK<br>VVLHPN#YSQVDIGLIK<br>MVSHHN#LTTGATLINEQWLLTTAK<br>GSFPWQAKMVSHHN#LTTGATLINEQWLLTTAK
57	O95714|HERC2_HUMAN	ubiquitin-protein	SLN#VSSSVNQASR<br>DAPHSEGDMHLLSGPLSPN#ESFLR
58.	O15297|PPM1D_HUMAN	Protein phosphatase	IHDSLNNSLPIGLVPTN#STNTVMDQK
59	O00141|SGK1_HUMAN	Serine/threonine-protein	RMGLNDFIQKIAN#NSYACK
60	F5HB53|GB_HCMVM	Envelope glycoprotein B	SSLN#LTHNRTK
61	B1NKQ9|RDRP_ROTH7	RNA-directed RNA polymerase	IIRVDGDDNYAVLQFNTEVTKQMVQN#VSNDVR
62	A8MTB9|CEA18_HUMAN	cell adhesion molecule	MN#LSSLAWEQMGRYR<br>VNREGSLLIRPTALN#DTGN#YTVR
63.	A4UHQ7|L_EBLV2	RNA-directed RNA polymerase	LLN#YTLENRGLAIPTDGVLSSLK
64	A1L390|PKHG3_HUMAN	Pleckstrin	NGAGSLRSRHLPNSNN#NSSSWLNVK<br>HLPNSNN#NSSSWLNVKGPLSPFNSR

© 2016 by the authors; licensee MDPI, Basel, Switzerland. This article is an open access article distributed under the terms and conditions of the Creative Commons by Attribution (CC-BY) license (http://creativecommons.org/licenses/by/4.0/).
